# Suppuration of the Antrum

**Published:** 1900-09-15

**Authors:** 


### SUPPURATION OF THE ANTRUM.

---

Dr. Corns makes report on antral case. Thinking that this case might interest you as it has me, I take this opportunity of reporting it. Dr. Preston M. Hickey, a throat and nose specialist, called me in consultation in a case he had been treating some time. A Mr. R., who complained of a severe pain, neuralgic in character, over the right eye and affecting the right cheek. At times he would be almost free from pain, which was immediately after profuse discharge from the right nostril, which led us to believe that it was a case of retained mucous secretions, and we decided to operate. I administered nitrous oxide gas and after complete anesthesia made an opening into the antrum at the location of the first molar; I used the dental engine with special large drill about one fourth of an inch in diameter. We found no discharge coming through the opening, but on syringing out the antrum with warm solution of glyco-thymoline a mucous discharge was forced through the opening into the nose. I then made a silver tube of the required length and placed it in position in the opening, then took an impression, which gave me the tube in position on the impression. From this I got a model. I put a clasp around the first bicuspid and soldered it to the tube.

The Result of Treatment—The case was operated upon April 27th; he is still under treatment and has never had any serious trouble since the opening was made into the antrum. He is a large man, and is very apt to take cold; simply riding on an open car he is likely to have an attack. It is still a little tender about cheek, but on cleaning out it is relieved at once. The mucous secretion is still coming out; it washes out through the tube. After syringing out about three syringefulls, then will come out this thick mucous, which is almost the consistency of a polypus.

Dr. Hickey thinks that before the case can be cured we will have to get a larger opening into the antrum. The case has got along as well as could be expected. Dr. Hickey has added a few words which I will read:

"At the request of Dr. Henry Corns I will add a few notes to the history of the case which the Doctor is bringing before you. Mr. R., aged fifty, was referred to me for an examination of the nose by one of my brother practitioners in the fall of 1899. He gave the following clinical history: For the last five years he had complained of a severe pain, neuralgic in nature, principally over the right eye and affecting also the right cheek. When the pain first came on he had thought that it had come from his teeth, and accordingly he had four teeth—the second bicuspid and three molars—on right upper jaw removed, although the dentist who made the extraction did not believe that they were the starting point of the pain. The attacks were paroxysmal in nature, occuring chiefly in winter and subsiding during the summer months. The pain was usually very severe, so as to prevent sleep, and was increased by mastication. Usually, when the pain was at its height, there would occur a mucous discharge from the right nostril, after which the severity of the attack would abate. At the time that I saw Mr. R. he had been unable to sleep or eat for nearly two weeks, spending the nights bolstered up in a chair. The attending physician had given him various anti-neuralgic remedies, and found that even heroic doses of the various anodines had very slight effect. Examination of the nasal passages showed nothing pathological as far as could be seen. However, a spray of 4 percent cocain, which was used for diagnostic purposes only, gave temporary relief much better than any of the anodine mixtures which had been used. Accordingly I ordered for him a spray of suprarenal extract for the purpose of keeping the nasal mucous membrane contracted and cleansed. It seemed to me at this time that the point of irritation was located in the antrum, but as the sprays of the suprarenal extract seemed to give some relief I cauterized very thoroughly the membrane over the inferior turbinated bone, so as to give as much room as possible for the escape of the pent up secretion. Mr. R. improved somewhat, but the attacks returned and I accordingly decided that it was advisable to open the antrum. Transillumination of the mouth by means of the electric lamp showed that there was no pus present in either antra. With the assistance of Dr. Corns the antrum was opened, the tube inserted which the Doctor will describe to you, and the cavity thoroughly irrigated. Opening the antrum did not reveal any pus, but upon syringing the antrum by means of the opening so that the fluid ran out through the natural opening, ostium maxillare, considerable inspissated mucous, of the consistency and appearance of the nasal polyp was brought out. Since the antrum was opened

Mr. R. has improved, and although not all of the time free from pain, still the attacks were at once aborted by cleansing out the antrum. Looking back at the case from the present standpoint, it would seem that the pathological condition had been one of retained mucous secretion, probably through the thickening of the membrane about the ostium maxillare. The solutions which have been employed for irrigation have been warm alkaline mixtures with occasional applications of a dilute iodin solution."

Dr. Oakman: I can not let this subject go by without having something to say. Dr. Corns speaks of using tube; I used this once in my practice, but have since discarded it. It is not possible to insert a tube so that it will be on a level with the floor of the antrum. In time the mucous membrane will close in over the tube and pus will not be carried away. The better way, I think, is to open into the antrum with a liberal opening. It must be large, because it takes some time to cure these cases. With a small tube you can not get the proper amount of antiseptic into the antrum. After cleaning out I pack with gauze. In removing the gauze from the antrum you know how much pus is present, because it is absorbed by the gauze and can be seen; with the tube it is constantly dripping in the mouth, you can not judge the amount, and it is unpleasant to the patient. I have in mind a case which a physician was treating, a bad case. After treating it for six months he said the patient was pretty nearly well and would not have to come and see him any more. I said, "It is all right, but what did you use?" "Salt and water and dilute glycerine, and he is nearly well." I saw the Doctor again and he informed me his patient was worse. I told him not to think of using salt, but to pack the antrum with gauze. Twenty-four hours after putting that in it was saturated with pus, and he is again coming for treatment.

If I have time I would like to tell of a very peculiar case which came under my observation. It was a young man twenty-four years of age; he was the electrician in agricultural college. He was assisting in some electrical work about the building and received a severe shock. It laid his skull bare along the side and over the ramus of the jaw on the left side of the face. He had received a shock of from 1,800 to 2,000 volts and he fell. He was taken to the hospital and while there in the course of three weeks he noticed a pain in the region of the left antrum. He had a tooth extracted after leaving the hospital some months later and still the pain did not cease, and a year passed by before I saw him. He had a very fetid breath, stomach seemed to be very much out of order and his color was anemic. When I informed him what the trouble was, he said he was not surprised. The members of his family had noticed his breath and thought he had catarrh. I made small opening at first to determine the amount of pus, found much there and very odoriferous. I used cocain hypodermic with ethyl chlorid, and enlarged opening. He was not in a condition to take a profound anesthetic; I was afraid of the shock. In this case I curetted the whole antrum. I think that nine times out of ten in these chronic cases it is the only thing to do.

